# Griscelli Syndrome Presented with Status Epilepticus and Hemophagocytic Lymphohistiocytosis

**DOI:** 10.4274/tjh.2015.0416

**Published:** 2017-03-01

**Authors:** Fatih Demircioğlu, Hilal Aydın, Mustafa Erkoçoğlu, Hüseyin Önay, Emine Dağıstan

**Affiliations:** 1 Abant İzzet Baysal University Faculty of Medicine, Department of Pediatrics, Division of Pediatric Hematology, Bolu, Turkey; 2 Abant İzzet Baysal University Faculty of Medicine, Department of Pediatrics, Division of Pediatric Neurology, Bolu, Turkey; 3 Abant İzzet Baysal University Faculty of Medicine, Department of Pediatrics, Division of Pediatric Immunology and Allergy, Bolu, Turkey; 4 Ege University Faculty of Medicine, Department of Molecular Biology and Genetics, İzmir, Turkey; 5 Abant İzzet Baysal University Faculty of Medicine, Department of Radiology, Bolu, Turkey

**Keywords:** children, Griscelli syndrome, Status epilepticus, Hemophagocytic lymphohistiocytosis

A 12-month-old female infant was referred to our hospital with prolonged fever and status epilepticus. Her weight and height were below the 5^th^ percentile for age. Physical examination revealed marked hypotonia, fever, pallor, partial albinism with silvery gray hair, and hepatosplenomegaly (Figure 1A). Laboratory investigations showed anemia, thrombocytopenia, hypofibrinogenemia, hyperferritinemia, and hemophagocytosis at bone marrow examination (Figure 1B). Lymphocyte subsets and serum immunoglobulin levels were normal. Hair examination showed irregularly scanty melanin pigments (Figure 1C). Electroencephalographic study revealed encephalopathic findings, including decreased background activity with continuous slow wave discharges. Brain magnetic resonance imaging showed diffuse cerebral involvement (Figure 2). RAB27A encoding gene C.149delG mutation was detected. We diagnosed Griscelli syndrome (GS) with hemophagocytic lymphohistiocytosis (HLH). She received the HLH-2004 treatment protocol. The patient showed complete hematological response to treatment and was discharged after 1 month with persistent neurological involvement. Although bone marrow transplantation is the only curative therapy for GS, we did not plan bone marrow transplantation due to the severe neurological sequela. The patient died due to progressive disease after 6 months.

GS is an autosomal recessive disorder characterized by the silvery gray sheen of the hair and hypopigmentation of the skin, which can be associated with neurological impairment, psychomotor retardation, HLH, and immunodeficiency [1]. Both GS and Chediak-Higashi syndrome may present with oculocutaneous albinism, neutropenia, immune dysfunction, and accelerated phase. In differential diagnosis, the absence of bleeding disorders and giant granules in leukocytes, and finally gene analysis, helped us to exclude Chediak-Higashi syndrome [2]. GS type 1 is caused by a mutation in the myosin Va (MYO5A) gene, GS type 2 is caused by mutations in the RAB27A encoding gene, and GS type 3 is due to mutations in the MLPH gene, which forms a protein complex with Rab27a and myosin Va [3,4]. Hematopoietic stem cell transplantation is the only curative treatment for GS with HLH [3,4].

## Figures and Tables

**Figure 1:**
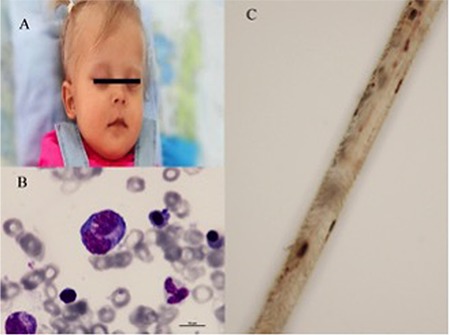
(a) Partial albinism with silvery gray hair. (b) Bone marrow examination showing hemophagocytosis. (c) Hair examination showing irregularly scanty melanin pigments.

**Figure 2:**
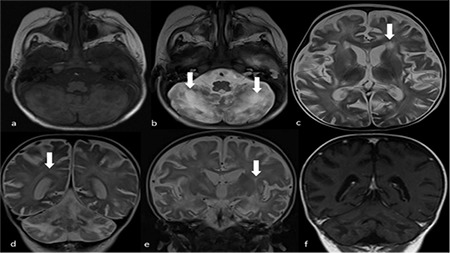
Griscelli syndrome: cerebral involvement. (a) Axial T1-weighted magnetic resonans (MR) image shows bilateral low-signal-intensity areas in white matter of cerebellum. (b) Axial fluid attenuation inversion recovery MR image demonstrates high-signal-intensity in this area. (c) Axial T2-weighted MR image at lateral ventricle level. (d, e) Axial and coronal T2-weighed images showing cerebral atrophy and diffuse high-signal-intensity in cerebral white matter. (f) Contrast-enhanced coronal T1-weighted MR image demonstrates no contrast uptake.
